# Study on carbon emission driving factors and carbon peak forecasting in power sector of Shanxi province

**DOI:** 10.1371/journal.pone.0305665

**Published:** 2024-07-12

**Authors:** Wei Hu, Tingting Zheng, Yi Zhang

**Affiliations:** College of Economics and Management, Shanghai University of Electric Power, Shanghai, China; Hong Kong Shue Yan University, HONG KONG

## Abstract

The realisation of the low-carbon transition of the energy system in resource-intensive regions, as embodied by Shanxi Province, depends on a thorough understanding of the factors impacting the power sector’s carbon emissions and an accurate prediction of the peak trend. Because of this, the power industry’s carbon emissions in Shanxi province are measured in this article from 1995 to 2020 using data from the Intergovernmental Panel on Climate Change (IPCC). To obtain a deeper understanding of the factors impacting carbon emissions in the power sector, factor decomposition is performed using the Logarithmic Mean Divisia Index (LMDI). Second, in order to precisely mine the relationship between variables and carbon emissions, the Sparrow Search Algorithm (SSA) aids in the optimisation of the Long Short-Term Memory (LSTM). In order to implement SSA-LSTM-based carbon peak prediction in the power industry, four development scenarios are finally built up. The findings indicate that: (1) There has been a fluctuating upward trend in Shanxi Province’s total carbon emissions from the power industry between 1995 and 2020, with a cumulative growth of 372.10 percent. (2) The intensity of power consumption is the main factor restricting the rise of carbon emissions, contributing -65.19%, while the per capita secondary industry contribution factor, contributing 158.79%, is the main driver of the growth in emissions. (3) While the baseline scenario and the rapid development scenario fail to peak by 2030, the low carbon scenario and the green development scenario peak at 243,991,100 tonnes and 258,828,800 tonnes, respectively, in 2025 and 2028. (4) Based on the peak performance and the decomposition results, resource-intensive cities like Shanxi’s power industry should concentrate on upgrading and strengthening the industrial structure, getting rid of obsolete production capacity, and encouraging the faster development of each factor in order to help the power sector reach peak carbon performance.

## 1. Introduction

One of the main industries contributing to greenhouse gas emissions is the energy sector [1]. More than 80% of China’s total emissions of carbon dioxide come from the energy sector, with the power sector contributing roughly 42.5% of these emissions [2, 3]. Future growth in energy consumption will mostly come from the power sector, which is the primary source of carbon emissions. The electricity industry has a higher potential for decarbonization and cost advantages in reducing carbon emissions than other sectors. Its emission reduction process and peak rate will be closely linked to the nation’s progress towards achieving the "double carbon" target [4, 5]. Since China’s regions have quite different levels of economic development, each province must design specific plans for reducing emissions based on its unique energy needs. Using Shanxi Province as an example, the rate of carbon peaking in its power sector has become the key to gauging the success of energy reform in Shanxi Province, which is the first and only pilot province for regional and comprehensive reform of China’s energy revolution [6–8]. Determining the future carbon peak and conducting a thorough analysis of the variables influencing carbon emissions in the power industry of Shanxi province is imperative. This holds significant value for constructing a contemporary energy system and advancing energy system reform, and it also offers valuable insights for other sizable energy regions.

Depending on the subject of research, different factors influence different carbon emissions. The choice of carbon emission factors is influenced by the study object’s duration, industry, and regional scope. In their investigation of the relationship between these factors and carbon emission, Shan et al. included fiscal decentralization, institutional quality, energy price, and GDP as carbon emission factors [9]. The findings indicated that there is an inverse U-shaped relationship between fiscal decentralization and carbon emission, and that increasing energy prices and institutional system quality will decrease carbon emission while increasing GDP will increase carbon emission. In their analysis of the factors influencing carbon emissions in the Philippines, Sumabat et al. discovered that rising living standards and economic expansion both greatly increase carbon emissions, a problem that is prevalent in developing nations [10]. In their analysis of the decarbonization of energy systems, Isik et al. showed that a rise in the proportion of fossil fuels has a notable effect on carbon emissions [11]. Finding a practical way to break down carbon emission variables is essential to lowering emissions. Using random forests, Qin et al. determined the major influencing factors and then assessed each factor’s significance in relation to carbon emissions based on its characteristic importance [12]. The advantages and disadvantages of each component, however, cannot be intuitively reflected by this approach. Using the extended STIRPAT model, Fang et al. examined the emission drivers of thirty Chinese provinces and projected carbon emissions from this data [13]. This article breaks down carbon emissions using the Logarithmic Mean Divisia Index (LMDI) approach. On the other hand, the LMDI model is more flexible because it can break down carbon emission drivers in a particular fashion and does not need that the functional link between the components and carbon emissions be known ahead of time.

Numerous researchers have used scenario analysis in conjunction with various prediction models to look at carbon emission patterns at the regional level in the study of carbon emission peak prediction. Wang et al. use the enhanced STIRPAT model to analyze the carbon emissions predicted from Shanghai’s energy industry [14]. Using the ARIMA model, Jia et al. simulate and predict the ecological footprint of Henan Province. This study validates the prediction model’s validity and gives managers a guide for organizing the region’s ecological balance for the long-term growth of the economy and society [15]. Related researchers use deep learning to address these kinds of issues. A hybrid prediction model combining LSTM and multi-intelligentsia was proposed by Bouziane et al. to forecast carbon emissions during the energy generation process [16]. Zuo et al. developed an integrated LSTM-STIRPAT prediction model to forecast the peak carbon emissions of 30 Chinese regions [17]. Because traditional deep learning models often overfit and have poor generalization capabilities, researchers have once again concentrated their efforts on increasing model accuracy. In this regard, Huo et al. employed GA to enhance the SVM model and more precisely forecast the Jiangsu province’s carbon emission trend across three scenarios [18]. The attention mechanism was presented by Lin et al., who also developed the Attention-LSTM prediction model to perform a prediction research of the undesirable carbon dioxide emissions outputs in many nations [19]. A review of the literature on carbon emission forecasting indicates that the majority of studies that use intelligent algorithms to optimize forecasting models are now focused on the transportation and construction sectors, with a dearth of research on the power sector.

Since its proposal, the objective of carbon peaking and carbon neutrality has drawn a lot of attention, and there are becoming more and more public discussions on the subject. A practical way to accomplish the dual-carbon goal is to investigate the factors that contribute to carbon emissions in important sectors and tailor carbon reduction strategies accordingly [20, 21]. Nonetheless, the majority of previous study has tended to concentrate on a wider variety of entities, including nations, continents, and synergistic areas. Additionally, due to variations in their respective development, cities have extremely distinct carbon emission sources and emission control tactics. When creating emission reduction plans, several major energy provinces should pay close attention to the unique characteristics of their own energy system. This research explores the techniques of energy-intensive cities for reducing emissions, with a particular focus on Shanxi Province [22–24]. Regarding the selection of research areas, there is a dearth of studies on carbon emissions related to the electric power sector in energy-structured cities, with the majority of research concentrating on transportation, manufacturing, building, etc [25–27]. This work aims to close this research gap by examining the carbon emissions from electric power in Shanxi province from three angles: time dimension, prediction technique improvement, and factor selection. In the selection of influencing factors, factors such as per capita industrial contribution value, energy consumption intensity, energy efficiency, etc. are innovatively introduced, and the contribution of carbon emissions is analysed from the dimensions of population, economy, technology and electricity. This work presents the sparrow search algorithm, which dynamically explores the LSTM network’s hyperparameters to find the global optimal solution. This effectively addresses the overfitting issue and enhances convergence rate and prediction accuracy in the context of prediction model selection. Furthermore, this paper relaxes the study year to the period of 1995–2020, which spans the period of economic system reform, structural strategic adjustment and comprehensive deepening reform in Shanxi Province. The results of the long-term study can more accurately reflect the regional peculiarities by thoroughly analyzing the elements that drive carbon emissions from the power sector in Shanxi Province. This helps to explain the emission reduction focus.

This paper’s framework is divided into four sections. The study’s history is provided in the first section, along with a dynamic overview of pertinent research techniques. Organizing the facts and methods used to realize the article is the second step. The third section examines the power sector’s emissions from 1995 to 2020 as well as the results of the influencing factors’ decomposition. Based on the decomposition, it then identifies the drivers that have the greatest impact, builds the SSA-LSTM prediction model using the four dimensions of electric power, technology, economy, and demographics, and applies scenario analysis to forecast trends. The research findings of this work are summed up in the fourth part. The final section offers emission reduction strategies for the energy-intensive region represented by Shanxi.

## 2. Materials and methods

### 2.1. Carbon emissions accounting for power sector

The methodology recommended by the Intergovernmental Panel on Climate Change (IPCC) is used in this study to calculate the electricity sector’s contribution to overall carbon emissions [28]. The accounting method calculates the carbon emissions consumed in the production process by accumulating, layer by layer, data on the activity levels of various fuels and parameters such as the corresponding emission factors.

C=AD×EH (1)

Where: *C* represents the CO_2_ emissions; *AD* represents the activity level contributing to the carbon emissions in the production or consumption process; and *EH* is the carbon emission coefficient associated with each unit of production or consumption activity.


AD=FC×NCV (2)

Among them, *FC* is the amount of fossil fuel consumed in production or consumption; *NCV* refers to the average low heating value of fossil fuels.

$$EH=CC\times OF\times 44/12=EFCO_{2}\times OF$$ (3)

Where: *CC* represents the carbon content of fossil fuels per unit of calorific value; *OF* represents the rate at which carbon in fossil fuels is oxidized; 44/12 represents the molecular mass ratio of carbon dioxide to elemental carbon; and *EFCO*_2_ represents the carbon dioxide emission factor.

### 2.2. LMDI decomposition model

The LMDI approach, proposed by scholar Ang in 1998, is a decomposition method that quantifies each impacting factor’s contribution to the target variable through exponential decomposition. The method is beneficial for dealing with data that includes zeros and negative numbers, and it does not produce residual terms. As a result, the LMDI approach has been extensively explored in studying the breakdown of influencing factors in the energy field [29, 30].


$$C=\frac{C}{E}\times \frac{E}{F}\times \frac{F}{T}\times \frac{T}{D}\times \frac{D}{G}\times \frac{G}{IS}\times \frac{IS}{P}\times P$$ (4)

Among them, *C* is the Shanxi power sector’s carbon emissions; *E* represents the total energy consumption; *F* is the thermal power generation; *T* denotes the entire power generation; *D* represents the total power consumption; *G* is the gross regional product; *IS* is the total output value of the secondary industry; and *P* indicates the number of permanent residents in Shanxi Province. The variables involved above are the minimum dataset for the study of this paper, which is relied upon for subsequent factor decomposition and peak prediction, as shown in Table 1. The experimental minimum data set’s descriptive statistics, including the mean, minimum, and maximum values, are displayed in Table 2.

**Table 1:** Minimum data set of factors influencing carbon emissions.

Year	Carbon emissions/tonnes	Population/million	Gross regional product/10^4^ CNY	Energy consumption/tonnes	Total output value of the secondary industry/ 10^4^ CNY	Thermal power generation/billion kWh	Total power generation/billion kWh	Electricity consumption/billion kWh
1995	5037.76	3077.28	10760300	28603900	4944500	498.85	505.97	399.16
1996	5331.21	3109.26	12921100	30365000	6002100	519.52	526.89	431.22
1997	5323.62	3140.89	14760000	30025200	7075800	540.26	546.02	446.01
1998	5369.93	3172.20	16110800	30191800	7612500	548.02	554.03	440.31
1999	5398.39	3202.63	16671000	30228400	7854700	558.45	569.83	459.34
2000	5550.08	3247.8	18457200	31273100	8583700	607.27	620.31	506.09
2001	6310.78	3271.63	20295300	35887000	9560100	691.14	710.33	557.08
2002	7429.73	3293.71	23248000	41729500	11343100	823.18	842.01	628.83
2003	8374.33	3314.29	28542500	47376200	15207300	945.71	965.01	731.77
2004	10042.85	3335.07	34959900	61361400	19306300	1058.00	1078.99	841.55
2005	11707.92	3355.21	40793800	82880528	23895100	1291.65	1311.97	892.46
2006	13603.57	3374.55	47136000	91323672	27992800	1502.50	1526.37	1036.06
2007	15050.57	3392.58	59355800	95792354	36023100	1713.18	1760.50	1269.69
2008	16106.66	3410.64	72229800	105723382	43889500	1764.93	1793.78	1238.25
2009	16657.72	3427.36	71476100	106268532	40912000	1848.76	1873.80	1196.24
2010	17909.42	3574.11	89039000	108815323	53496400	2104.00	2151.00	1381.25
2011	19460.40	3562.37	108944100	118473448	67501500	2301.73	2343.97	1576.23
2012	20488.06	3548.21	116831100	124623103	68527000	2456.14	2545.91	1672.39
2013	21793.38	3534.98	119872300	125720787	66843200	2551.32	2641.11	1731.48
2014	20724.59	3528.49	120947100	126221203	63779600	2546.03	2647.02	1726.14
2015	18438.26	3518.62	118363900	124151935	52196500	2330.26	2449.00	1644.09
2016	17964.01	3514.48	119464000	127100909	51136200	2362.85	2535.00	1706.94
2017	20924.11	3510.46	144842700	128289160	66353300	2607.22	2861.00	1893.59
2018	22521.87	3502.47	159581300	126479434	70744600	2853.44	3203.00	2175.43
2019	22862.42	3496.88	169616100	132648156	74663000	2960.80	3362.00	2233.63
2020	23783.14	3490.50	176519300	131285167	76754400	3032.50	3504.00	2343.49

**Table 2:** Table of descriptive statistics for the smallest data set.

Variable	C	P	G	E	IS	F	T	D
Max	23783.14	3574.11	176519300	132648156	76754400	3032.5	3504	2343.49
Min	5037.76	3077.28	10760300	28603900	4944500	498.85	505.97	399.16
Mean	14006.34	3381.03	74297635	85493792	37776857.69	1654.53	1747.26	1198.41

Further, *CE* = *C*/*E* represents the intensity of the energy structure; *EF* = *E*/*F* is the energy consumption intensity of thermal power; *FT* = *F*/*T* means the power supply structure; *TD* = *T*/*D* denotes the conversion rate of electricity generation; *DG* = *D*/*G* represents the power consumption intensity; *GI* = *G*/*IS* is the reciprocal of industrial structure; *IP* = *IS*/*P* represents the per capita secondary industry value. Therefore, formula (4) can be expressed as:

C=CE×EF×FT×TD×DG×GI×IP×P (5)

Using the additive decomposition form of LMDI, Eq (5) is decomposed to obtain the following equation:

$$\Delta C=C^{T}-C^{0}=\Delta C_{CE}+\Delta C_{EF}+\Delta C_{FT}+\Delta C_{TD}+\Delta C_{DG}+\Delta C_{GI}+\Delta C_{IP}+\Delta C_{P}$$ (6)

Where: *C*^*T*^ and *C*^0^ represent the carbon emissions at the end and base period, respectively; Δ*C*_*CE*_ denotes energy structure effect; Δ*C*_*EF*_ is thermal power energy consumption intensity effect; Δ*C*_*FT*_ indicates power structure effect; Δ*C*_*TD*_ represents electricity generation and consumption conversion rate effect; Δ*C*_*DG*_ denotes electricity consumption intensity effect; Δ*C*_*GI*_ is industrial structure effect; Δ*C*_*IP*_ means per capita secondary industry contribution effect; Δ*C*_*P*_ represents population size effect.


$$W=\frac{C^{T}-C^{0}}{\text{ln}\;C^{T}-\text{ln}\;C^{0}}$$ (7)

$$\Delta C_{CE}=W\times \text{ln}\;\frac{CE^{T}}{CE^{0}}$$ (8)

$$\Delta C_{EF}=W\times \text{ln}\;\frac{EF^{T}}{EF^{0}}$$ (9)

$$\Delta C_{FT}=W\times \text{ln}\;\frac{FT^{T}}{FT^{0}}$$ (10)

$$\Delta C_{TD}=W\times \text{ln}\;\frac{TD^{T}}{TD^{0}}$$ (11)

$$\Delta C_{DG}=W\times \text{ln}\;\frac{DG^{T}}{DG^{0}}$$ (12)

$$\Delta C_{GI}=W\times \text{ln}\;\frac{GI^{T}}{GI^{0}}$$ (13)

$$\Delta C_{IP}=W\times \text{ln}\;\frac{IP^{T}}{IP^{0}}$$ (14)

$$\Delta C_{P}=W\times \text{ln}\;\frac{P^{T}}{P^{0}}$$ (15)

### 2.3. Carbon emission model of power sector in Shanxi Province based on SSA-LSTM

#### 2.3.1. Variable selection

The positive and negative drivers with the top three carbon emission contribution values were chosen by the decomposition results. The secondary industry’s gross output value, the gross regional product, the population, total energy consumption, carbon emissions in the power sector, electric power consumption, thermal power generation, and total power generation are the variables that make up these drivers. Based on this, the power sector carbon emission model in this study is built using eight different types of variables from the four dimensions of population size, economics, technology, and power development level. Table 3 lists the particular variables and their descriptions [31].

**Table 3:** Description of model variables.

Dimension	Variable	Data specification
Size of population	Population	The year-end resident population of Shanxi Province
Economics	Per capita GDP	GDP /Year-end resident population
	Industrial structure	Secondary value added/GDP
Technical	Carbon emission intensity	Carbon emissions/GDP from the power sector
	Energy intensity	Energy consumption/GDP
Level of power development	Thermal power contribution generation	Thermal power generation/total power generation
	Electricity consumption intensity	Electricity consumption/GDP

#### 2.3.2. LSTM model

**Fig 1:**
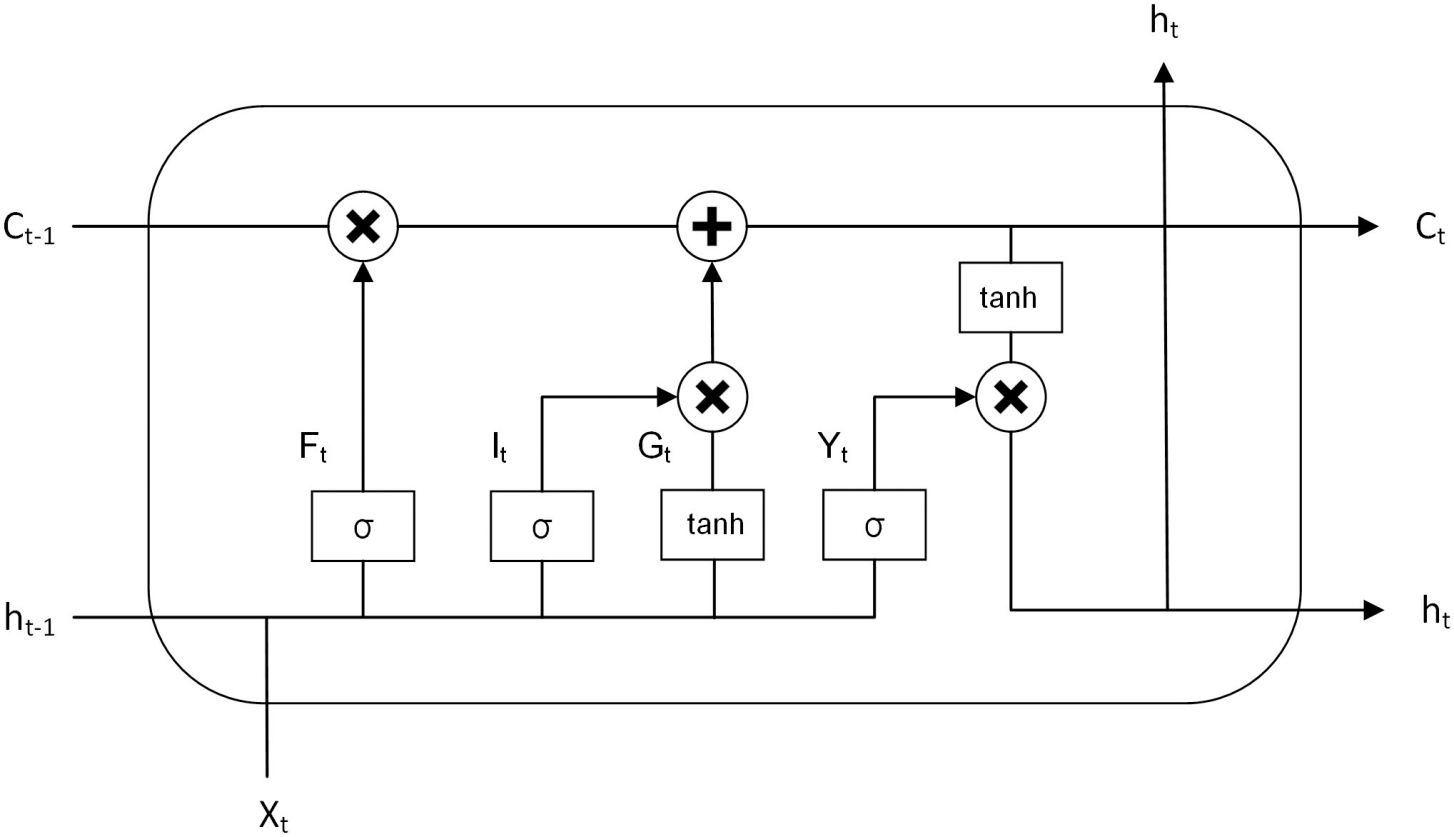
Structure of LSTM model.

The development of LSTM began with the recurrent neural network (RNN), which was then built upon to include forgetting gates, input gates, and output gates. Adding storage units also helped address the gradient vanishing and gradient explosion issues [32]. Fig 1 is the network structure diagram:

First, the information *h*_*t*−1_ of the previous state is selectively forgotten through a forgetting gate. Inputting *h*_*t*−1_ and the input information *x*_*t*_ of the current state into the sigmoid function *σ*, the output *F*_*t*_ is:

$$F_{t}=\sigma (W_{f}\times [h_{t-1},x_{t}]+b_{f})$$ (16)

Second, the information is selectively memorized through input gates, combining tanh and sigmoid layers to update the cell state:

$$I_{t}=\sigma (W_{i}\times [h_{t-1},x_{t}]+b_{i})$$ (17)

$$G_{t}=\text{tanh}(W_{g}\times [h_{t-1},x_{t}]+b_{g})$$ (18)

Further, the LSTM network updates the state of the old cells:

$$C_{t}=F_{t}\times C_{t-1}+I_{t}\times G_{t}$$ (19)

Finally, the final output of the cell unit is updated through the output gate:

$$Y_{t}=\sigma (W_{y}\times [h_{t-1},x_{t}]+b_{y})$$ (20)

$$h_{t}=Y_{t}\times \text{tanh}(C_{t})$$ (21)

Where: *W* is the weight; *b* represents the deviation matrix; *t* is the moment; *C* denotes the cell state; *σ* is the sigmoid activation function; tanh is the activation function.

#### 2.3.3. SSA model

As a population intelligent optimization algorithm with the ability of global search and local exploitation, the sparrow search algorithm divides the population into discoverers and joiners and searches for optimization in different dimensions by mimicking the characteristics of a sparrow population [33]. Assuming that there are n sparrows and m parameter dimensions to be optimized in the population, the population can be expressed as:

$$X=\left[\begin{array}{cccc} x_{11} & x_{12} & \cdots & x_{1m}\\ x_{21} & x_{22} & \cdots & x_{2m}\\ \vdots & \vdots & \vdots & \vdots \\ x_{n1} & x_{n2} & \cdots & x_{nm} \end{array}\right]$$ (22)

The discoverer has a wider search area and is tasked with finding food. Eq (23) is the location update formula for each iteration of the discoverer.

$$X_{ij,t+1}=\left\{\begin{array}{c} X_{ij,t}\cdot \text{exp}(-\frac{i}{\alpha \cdot i_{item,\text{max}}}),R_{2}<S_{ST}\\ X_{ij,t}+Q\cdot L,R_{2}\geq S_{ST} \end{array}\right.$$ (23)

Where: *t* is a representation of the current iteration count; *i*_*item*, *max*_ indicates the maximum number of iterations; *X*_*if*_ is the sparrow’s location within the dimension; *α* ∈ [0, 1] denotes a random number; *R*_2_ ∈ [0, 1] is the warning value; *S*_*ST*_ ∈ [0.5, 1] is the safety value; *Q* is a random number that satisfies the standard normal distribution; the values of the elements in the matrix are all 1.

Eq (24) is the location update formula for each iteration of the joiner.


$$X_{ij}^{t+1}=\left\{\begin{array}{c} Q\cdot \text{exp}(\frac{X_{worst}-X_{ij}^{t}}{i^{2}}),i>n/2\\ X_{p}^{t+1}+\left|X_{ij}^{t}-X_{P}^{t+1}\right|\cdot A^{+}\cdot L,i\leq n/2 \end{array}\right.$$ (24)

Among them, *X*_*p*_ denotes the discoverer’s best position.; *X*_*worst*_ is the worst place; *A* is a 1 × *d* matrix, and each matrix member is randomly assigned a value of 1 or -1, *A*^+^ = *A*^*T*^ (*AA*^*T*^)^−1^.

The 10–20% of the sparrow population can recognize the danger, and this portion of the sparrow individuals and their locations are randomly generated with the expression of Eq (25).

$$X_{ij}^{t+1}=\left\{\begin{array}{c} X_{best}^{t}+\beta \cdot \left|X_{ij}^{t}-X_{best}^{t}\right|,f_{i}>f_{g}\\ X_{ij}^{t}+K\cdot (\frac{\left|X_{ij}^{t}-X_{best}^{t}\right|}{(f_{i}-f_{w})+\varepsilon }),f_{i}=f_{g} \end{array}\right.$$ (25)

Where: *X*_*best*_ is the global optimal position of the current state; *β* is a random value and obeys a normal distribution with mean 0 variance 1; K is a random number in the range -1 to 1; *f*_*i*_ stands for the individual sparrow’s fitness value; *f*_*g*_ is the highest overall fitness value; *f*_*w*_ is the globe’s least favorable fitness value; *ε* is a very tiny constant.

#### 2.3.4. Combination of SSA and LSTM model

**Fig 2:**
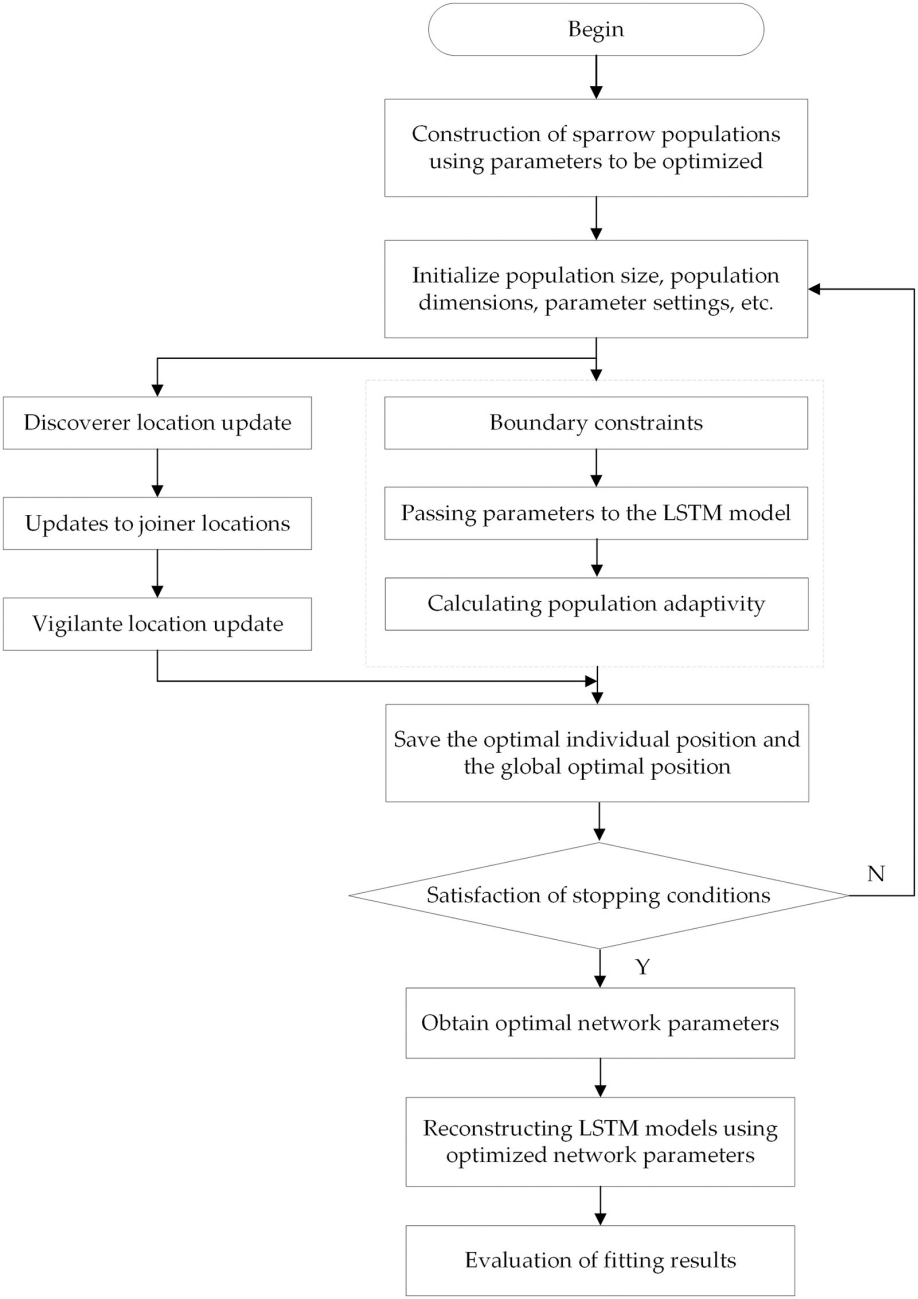
The steps of SSA-LSTM modelling.

The benefits of LSTM include efficient time-series data feature extraction and a brief training period. Nonetheless, the choice of an LSTM network’s hyperparameters has a significant impact on how accurate the network predicts. Because the globally optimal state of the network is rarely reached by the empirically chosen network parameters, it is challenging to guarantee the network’s ideal state. The two primary areas of LSTM improvement are parameter optimization and network architecture. By deepening the network, CNN-LSTM, Bi-LSTM, and DLSTM are meant to enhance model performance [34]. This study presents a model that combines the SSA optimization approach and LSTM features. It investigates the relationship between variables and carbon emissions in detail and dynamically finds the ideal number of iterations, implicit layer node count, and LSTM learning rate. Fig 2 shows the overall structure of SSA-LSTM. Here are the main steps of the proposed approach:

1. Model initialization: We start by determining the optimization parameters of the LSTM network. We also set the population size and initial position for the SSA model, the maximum number of iterations, and the upper and lower limits for the parameter values.
2. Objective function: The root mean square error (RMSE) between the initial untrained LSTM model’s predicted and actual values is what we refer to as the SSA model’s objective function.
3. Optimization: Based on the results of the objective function, we update the positions of the discoverers, joiners, and danger recognizers in real-time. This allows us to find the optimal parameter values when the initially set number of iterations is reached.
4. LSTM training: Once we have obtained the optimal parameters, we input them into the LSTM network and retrain the model. This allows us to derive an optimized prediction model.
5. Performance evaluation: We evaluate the accuracy and effectiveness of the SSA-LSTM network model using metrics such as the root mean square error (RMSE) and the mean absolute error (MAE).

### 2.4. Data sources

The "Energy Balance Sheet of Shanxi (Physical Quantity)" in the China Energy Statistical Yearbook provided the information needed to calculate Shanxi Province’s carbon emissions in the power sector from 1995 to 2020 [35]. There are 18 different energy variations overall that are used to quantify carbon emissions, including raw coal, washed coal, coal, etc. The total energy consumption, thermal power generation, and electric power consumption needed for the research of influencing factors are provided by the China Energy Statistical Yearbook. The Shanxi Statistical Yearbook for 2021 provides data on population, GDP, secondary industrial production, and overall population. The China Energy Statistical Yearbook provides data on all forms of energy generation.

## 3. Results

### 3.1. Carbon emissions from the power sector in Shanxi Province

**Fig 3:**
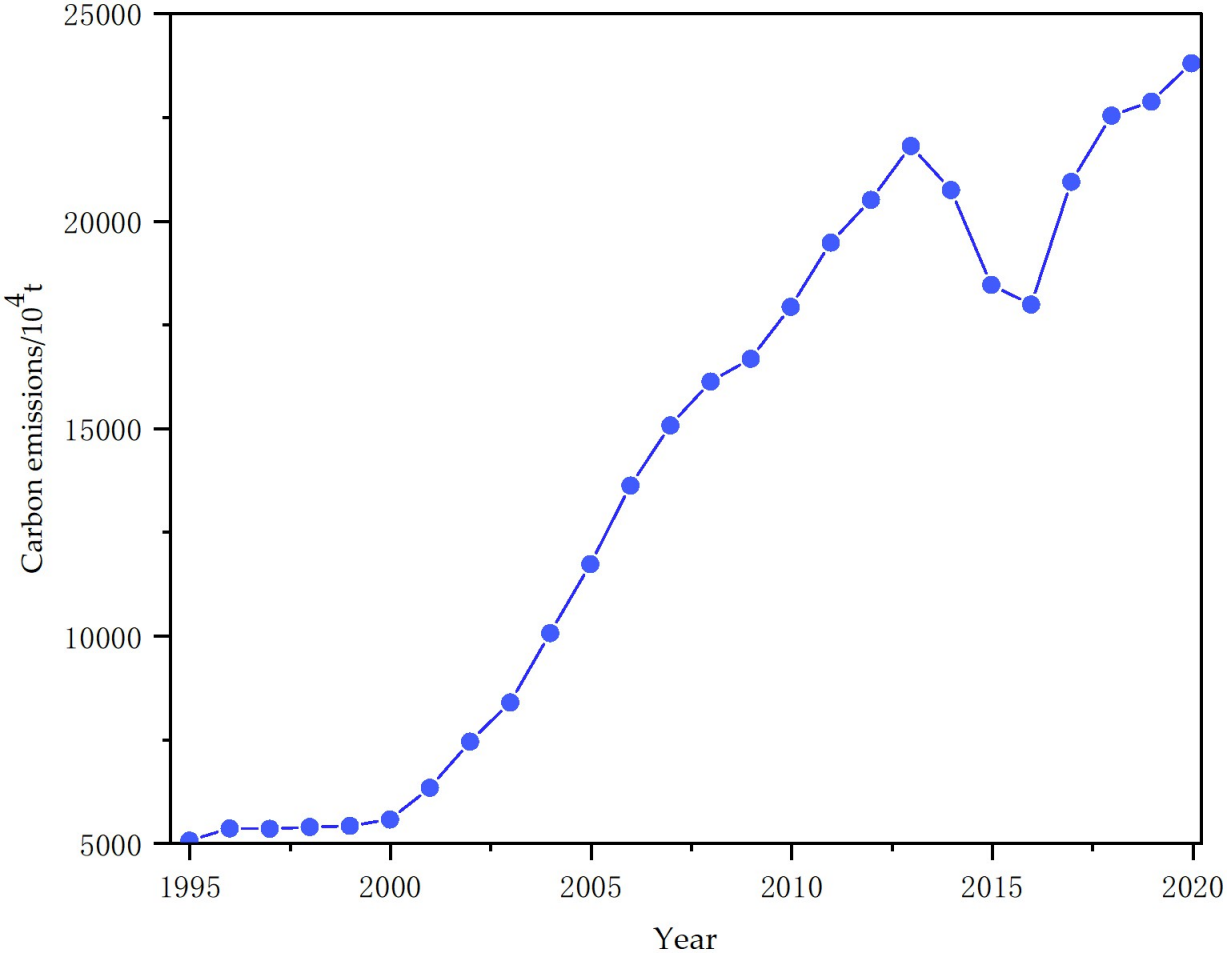
Carbon emissions from power sector in Shanxi Province, 1995–2020.

According to Eq (1) carbon emissions can be calculated for 1995–2020. As depicted in Fig 3, the carbon emissions exhibited a general upward trend throughout the study period. Notably, there was a significant increase from 2005 to 2013, and a turning point occurred in 2013 when the emissions reached 217,933,700 tonnes. Since then, there has been a decrease. However, from 2017 to 2020, the emissions continued to rise, albeit at a slower rate. This indicates that the power industry in Shanxi Province has been successful in implementing low-carbon transformations.

### 3.2. Driving factors of carbon emissions

**Fig 4:**
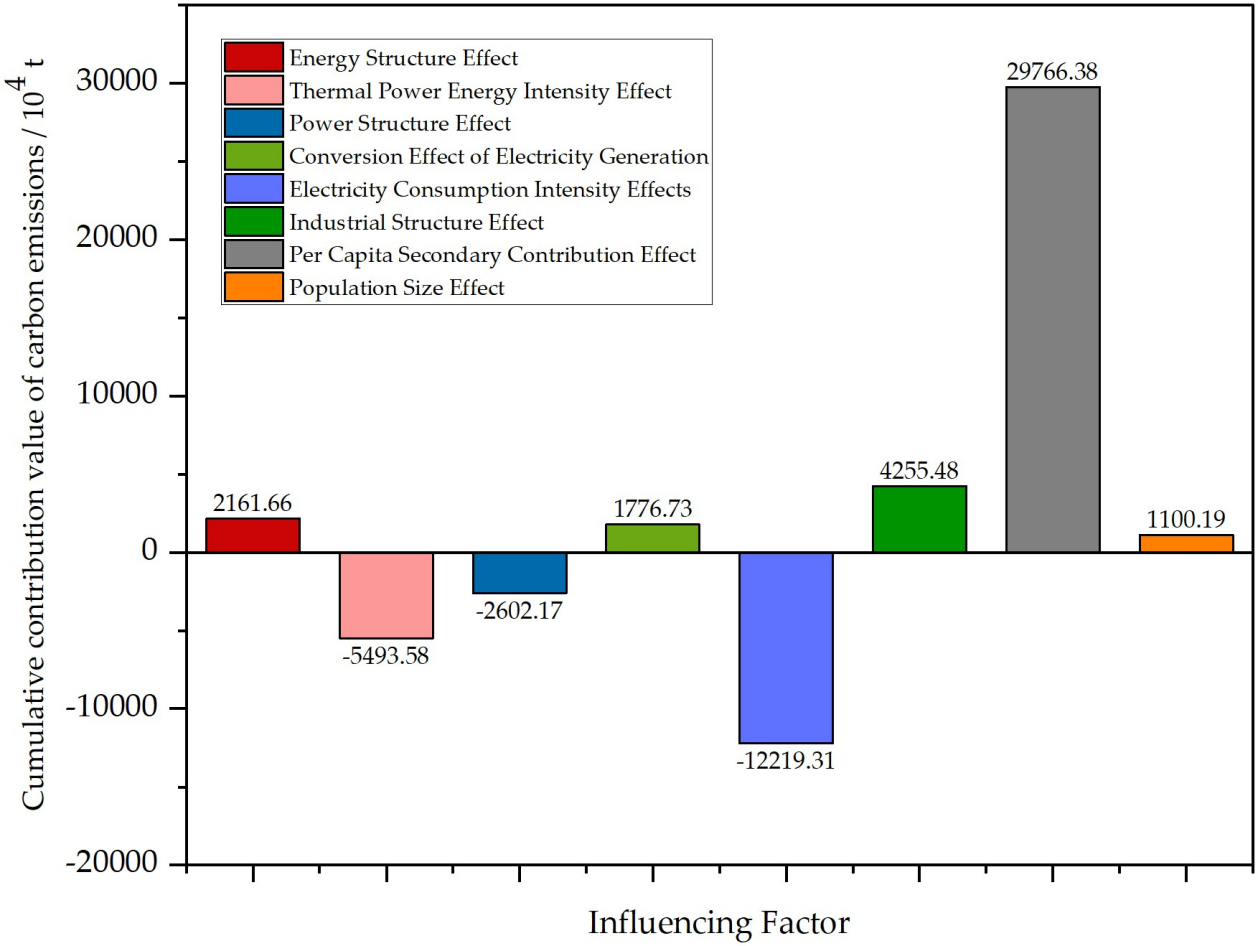
Cumulative contribution of carbon emissions from each factor.

This paper uses the LMDI model to break down the carbon emissions of the electric power industry in Shanxi Province from 1995 to 2020 in order to better understand the driving factors influencing changes in carbon emissions from electric power. Fig 4 displays the cumulative contribution values of each factor.

From the decomposition results, It is evident that the influencing factors that positively drive the carbon dioxide emissions are, in descending order of influence, the per capita secondary industry contribution effect, the industrial structure effect, the energy structure effect, the generation and use of electricity conversion rate effect, and the population scale effect. Among them, the positive driving impact of per capita secondary industry contribution efficiency is in a fault-type leading position because Shanxi Province, an underdeveloped coal resource-based representative province in the central region of China, has an imbalance in the development of the coal-related industrial sector. The pillar industry is single and crude, which has become a key constraint to the sustainable development of the economy of Shanxi Province. The influencing factors that negatively drive CO_2_ emissions are, in descending order of inhibition, the power consumption intensity effect, the thermal power energy consumption intensity effect, and the power supply structure effect. For a comprehensive investigation to examine the patterns in the influences of the variables throughout time. The drivers’ contribution values are broken down year by year in Table 4.

**Table 4:** A breakdown of the contribution of each factor [36].

Year	Energy Structure Effect	Thermal Power Energy Intensity Effect	Power Structure Effect	Generation-to-Electricity Conversion Rate Effect	Electricity Consumption Intensity Effects	Industrial Structure Effect	Per Capita Secondary Contribution Effect	Population Size Effect	Total Effect
1995–1996	-16.23	99.24	0.44	-190.44	-548.07	-56.16	951.07	53.59	293.45
1996–1997	52.36	-268.50	18.55	10.34	-529.21	-167.88	822.82	53.92	-7.59
1997–1998	16.72	-46.67	-1.61	146.64	-536.98	77.30	337.87	53.04	46.31
1998–1999	21.94	-94.99	-49.89	-76.41	43.78	15.40	117.23	51.40	28.46
1999–2000	-34.30	-272.77	-5.87	-65.92	-26.60	71.33	409.16	76.66	151.69
2000–2001	-54.30	48.85	-36.37	234.03	6.27	-75.80	594.73	43.29	760.70
2001–2002	84.98	-164.52	32.70	335.28	-100.62	-241.17	1126.18	46.11	1118.95
2002–2003	-57.06	-93.52	19.06	-120.43	-422.81	-694.48	2264.68	49.16	944.60
2003–2004	-706.86	1345.00	5.12	-258.39	-578.81	-329.21	2134.26	57.40	1668.52
2004–2005	-1597.88	1097.12	43.80	1484.50	-1037.57	-639.45	2249.20	65.35	1665.07
2005–2006	670.22	-684.66	-1.93	27.33	59.27	-173.91	1926.72	72.60	1895.65
2006–2007	763.14	-1194.55	-164.40	-868.09	-388.87	-310.64	3534.12	76.28	1447.00
2007–2008	-480.05	1072.70	171.80	682.10	-3447.42	-18.87	2993.15	82.68	1056.09
2008–2009	466.81	-675.88	45.22	1280.30	-393.56	978.94	-1230.87	80.11	551.06
2009–2010	842.56	-1825.08	-149.25	-100.91	-1311.35	-837.53	3908.96	724.31	1251.70
2010–2011	-37.01	-89.34	72.97	-861.51	-1301.84	-574.70	4403.85	-61.44	1550.98
2011–2012	17.08	-286.06	-353.71	467.78	-213.21	1094.67	380.64	-79.54	1027.66
2012–2013	1119.99	-618.14	27.65	42.00	190.74	1068.87	-446.83	-78.95	1305.32
2013–2014	-1153.22	128.52	-91.61	113.18	-255.37	1186.90	-958.12	-39.06	-1068.79
2014–2015	-1963.02	1408.76	-211.25	-568.27	-530.27	3497.73	-3865.22	-54.79	-2286.33
2015–2016	-901.51	174.48	-375.38	-54.63	514.41	541.89	-352.09	-21.43	-474.25
2016–2017	2779.52	-1729.32	-437.84	333.90	-1724.48	-1317.07	5077.61	-22.21	2960.10
2017–2018	1906.24	-2267.90	-492.37	-560.96	908.62	712.68	1440.92	-49.48	1597.76
2018–2019	-740.04	242.49	-261.27	500.27	-784.73	160.56	1259.52	-36.25	340.55
2019–2020	1161.58	-798.85	-406.73	-154.94	189.37	286.05	686.82	-42.59	920.73

**Fig 5:**
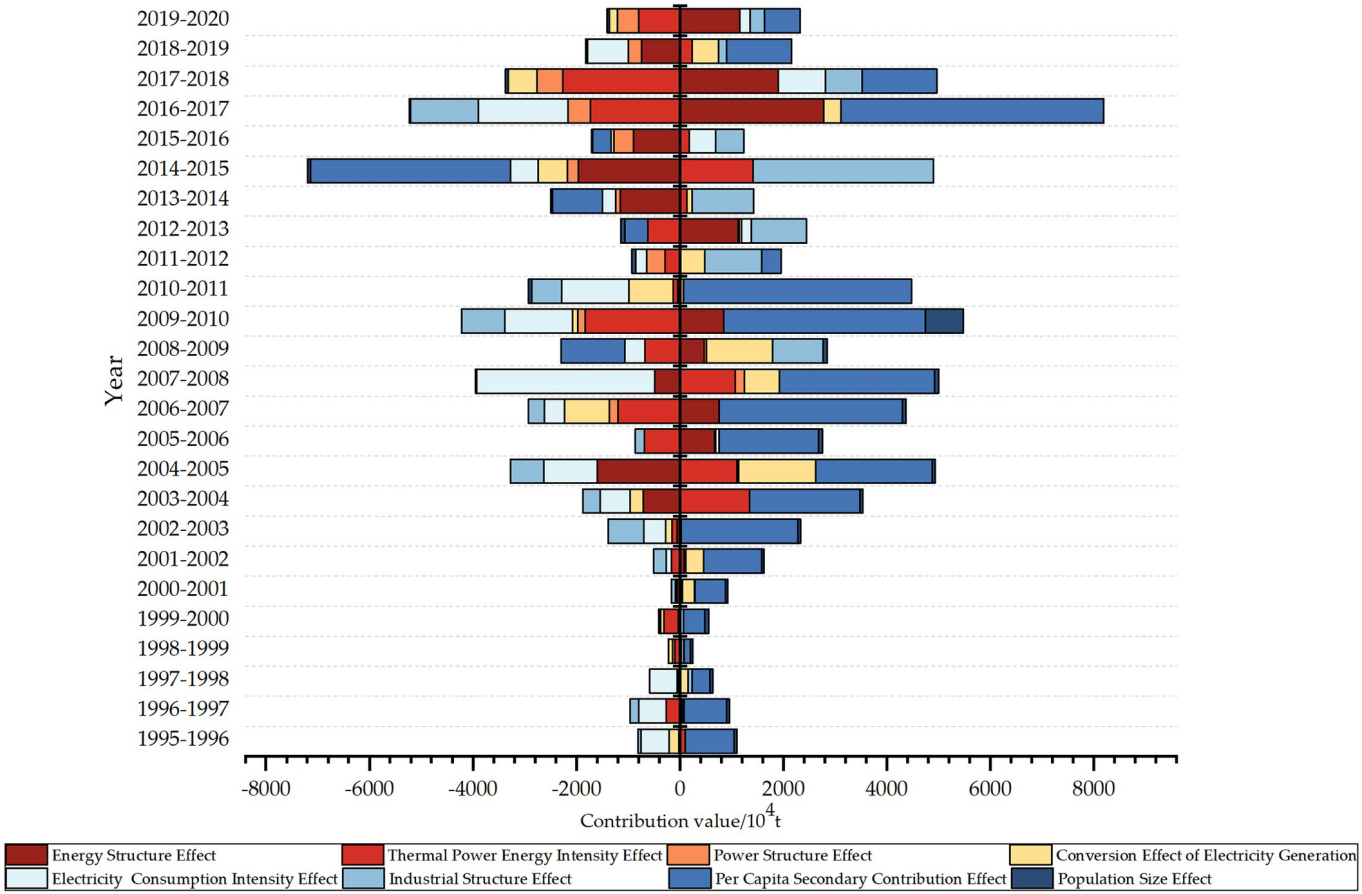
Decomposition of the value of contributions.

Fig 5 depicts the results of decomposing the contribution value of carbon dioxide emissions by influencing factors yearly. The decomposition results reveal that the per capita contribution factor of the secondary industry, the industrial structure factor, and the energy structure intensity factor all contribute significantly. The contribution of the per capita secondary industry, as the primary influencing factor in promoting carbon emissions, reflects Shanxi Province’s level of regional economic development and the secondary industry’s economic efficiency. The year-by-year decomposition results clearly show that this factor will increase carbon emissions for ten years, from 1995 to 2020. Since there was relatively little demographic change during the study period, the factor that played an important role in determining this factor was the total value of the secondary sector. The industrial structure factor, as the second-largest element positively impacting carbon emissions, has a positive driving role for nine years during the research phase. The energy structure intensity factor, representing the carbon emission intensity per unit of thermal power energy consumption, has played a driving role for eight years, reflecting the rationality of the energy structure. The findings of the analysis show that the total value of the secondary industry, GDP, and the resident population are critical factors in promoting carbon emissions. To effectively reduce carbon emissions, Shanxi Province must focus on industrial restructuring, promoting industrial transformation and upgrading. Additionally, the energy structure intensity is a significant element influencing carbon emissions, necessitating a focus on adjusting the energy structure and developing a new kind of electric power system to expedite the emission reduction process.

Electricity consumption intensity, thermal power energy intensity and power structure have a remarkable negative driving function. The primary factor is the power consumption intensity effect, which reflects electricity consumption’s efficiency. As electricity consumption per unit of regional GDP decreases, electricity production also declines, leading to reduced carbon emissions. The year-by-year decomposition chart from 1995–2020 shows that the electricity consumption intensity effect has reduced carbon emissions in eighteen years. The thermal power energy intensity impact, which measures energy consumption per unit of thermal power generation, has reduced carbon emissions in sixteen years during the study period. However, its contribution is slightly smaller than the power consumption intensity effect. The power structure has reduced carbon emissions in fifteen years. To address these issues, Shanxi Province should take action to manage power use, increase electricity use efficiency, and promote coordination between the regional economic scale, electricity consumption level, and resource environment. Additionally, optimizing fuel conversion technology and enhancing energy consumption conversion efficiency is crucial. Furthermore, in order to strengthen the proportion of renewable energy generation and actively pursue sustainable development, there should also be an emphasis on boosting the installation of renewable energy resources like wind power and photovoltaic.

### 3.3. Carbon emission prediction

#### 3.3.1. Setting parameters and analyzing model comparisons

**Fig 6:**
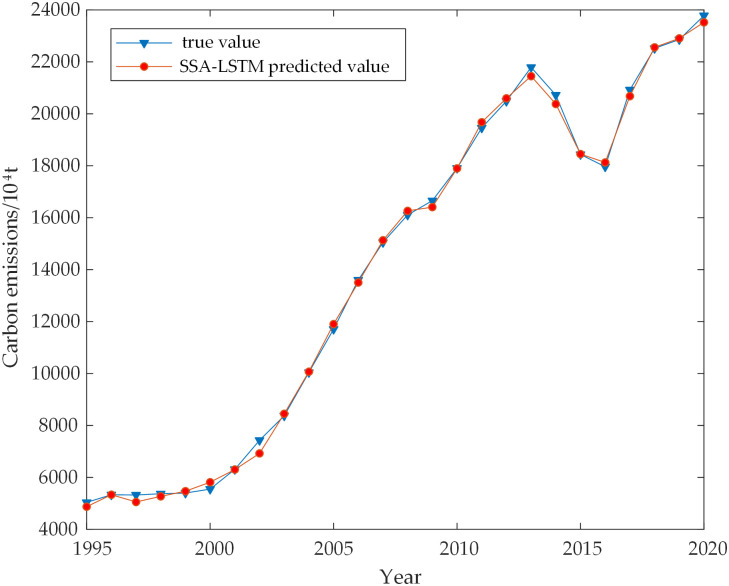
Comparison of SSA-LSTM predicted and true values.

**Fig 7:**
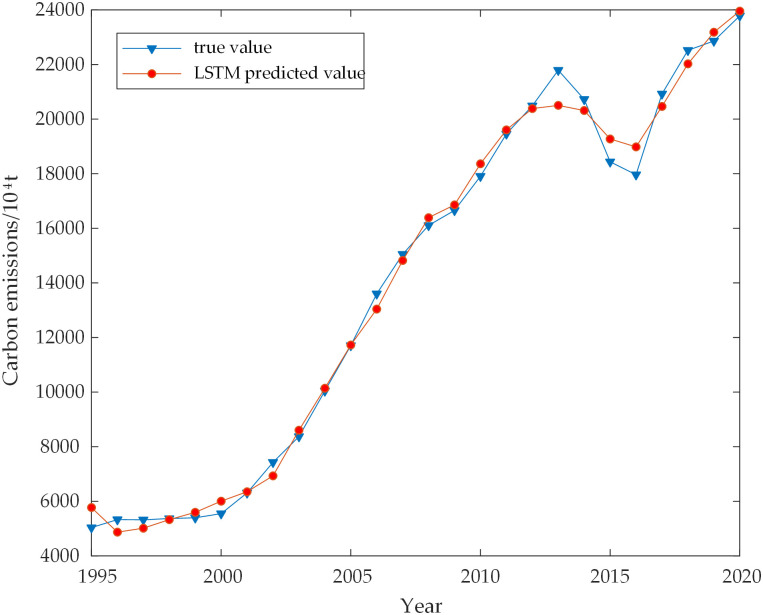
Comparison of LSTM predicted and true values.

**Fig 8:**
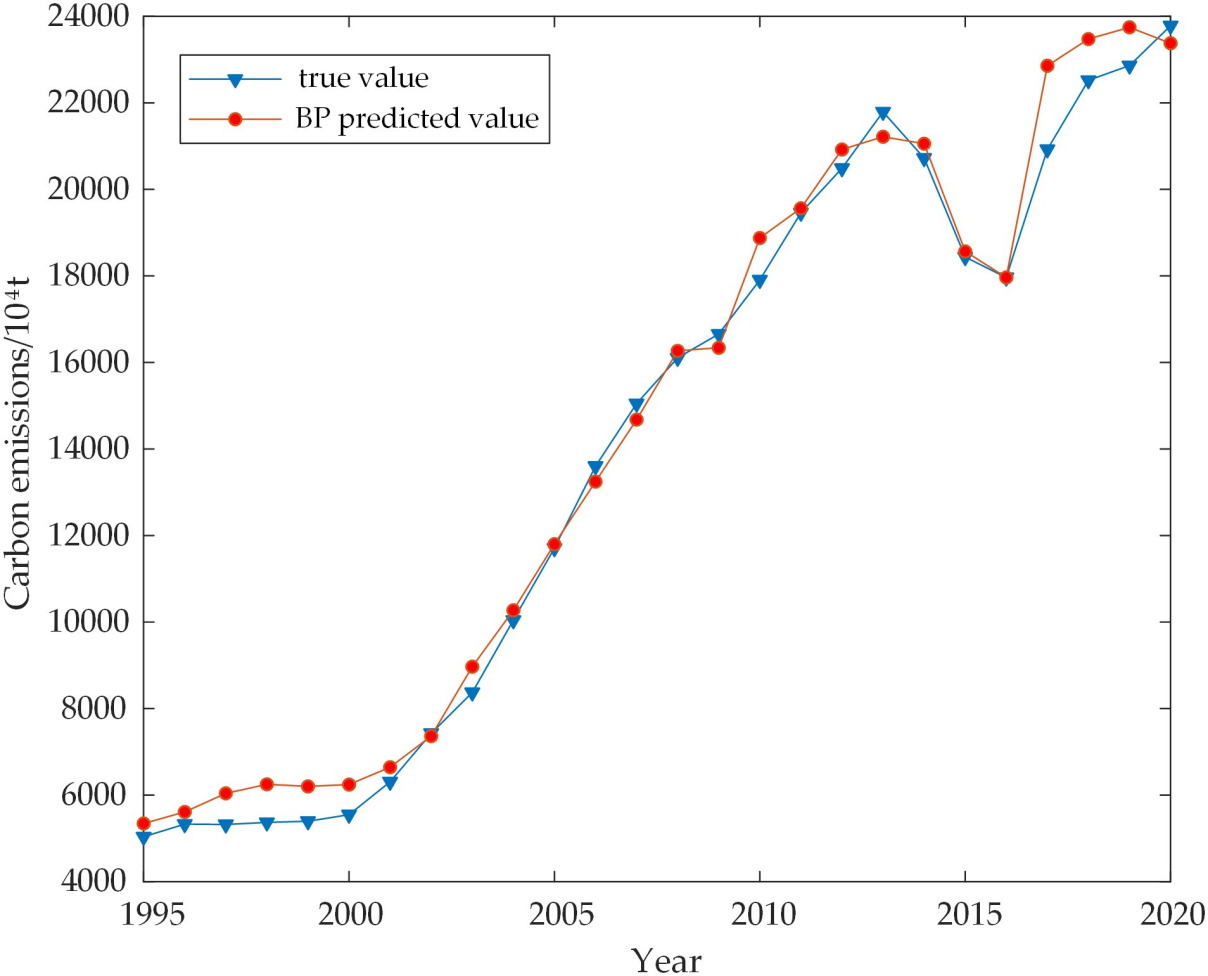
Comparison of BP predicted and true values.

The direct nonlinear relationship between each influencing factor and carbon emission was mined using the SSA-LSTM model. The hyperparameters of the LSTM network were optimized using the SSA model. The hidden layer’s ideal number of neuron nodes was 10, with a learning rate of 0.046 and a maximum amount of iterations of 75. The model was fitted to the actual carbon emissions data from 1995 to 2020, resulting in a coefficient of determination of 99.911, indicating a high level of accuracy. The results are in Fig 6. To assess the accuracy of the SSA-LSTM model visually, the sequence data were also input into the LSTM network and BP network. Figs 7 and 8 depict the prediction findings. It is noticeable from the figures that the LSTM model performs poorly at certain inflection points, while the BP model exhibits inaccuracies in both data trends and overall accuracy. In comparison, the SSA-LSTM model outperforms both models regarding error, as indicated in Table 5.

**Table 5:** Comparison of the prediction accuracy of the three models SSA-LSTM, LSTM, and BP.

Results	Model Category		
	SSA-LSTM	LSTM	BP
MAE	159.37	387.27	407.82
RMSE	201.49	491.89	579.96

#### 3.3.2. Scenario settings

The scenario analysis approach is utilized to examine the development of various aspects of the region in multiple scenarios. Using historical data from 1995 to 2020 in Shanxi Province, this study establishes parameter change rates under four scenarios—green development scenario, low carbon scenario, baseline scenario, and rapid development scenario—based on the current state of economic growth. As in Table 6, considering the requirements of the "14th Five-Year Plan" and the impact of the COVID-19 pandemic, seven independent variables are analyzed.

**Table 6:** Parameter settings for different scenarios.

Scene	Parameters	2021–2025	2026–2035	2036–2045
Low carbon scenarios	Population	0.08	0.01	-0.04
	Per capita GDP	6	5.5	4
	Carbon emission intensity	-5.4	-5	-4.6
	Energy intensity	-3.5	-4	-4.5
	Industrial structure	-4.3	-4	-3.7
	Thermal power contribution generation	-3.8	-3.4	-3
	Electricity consumption intensity	-2.6	-2.2	-1.8
Green development scenarios	Population	0.06	-0.01	-0.06
	Per capita GDP	5.5	5	3.5
	Carbon emission intensity	-4.9	-4.5	-4.1
	Energy intensity	-3	-3.5	-4
	Industrial structure	-4.1	-3.8	-3.5
	Thermal power contribution generation	-3.3	-2.9	-2.5
	Electricity consumption intensity	-2.3	-1.9	-1.5
Baseline scenario	Population	0.08	0.01	-0.04
	Per capita GDP	6	5.5	4
	Carbon emission intensity	-4.9	-4.5	-4.1
	Energy intensity	-3	-3.5	-4
	Industrial structure	-4.1	-3.8	-3.5
	Thermal power contribution generation	-3.3	-2.9	-2.5
	Electricity consumption intensity	-2.3	-1.9	-1.5
Rapid development scenario	Population	0.1	0.03	-0.02
	Per capita GDP	6.5	6	4.5
	Carbon emission intensity	-4.4	-4	-3.6
	Energy intensity	-2.5	-3	-3.5
	Industrial structure	-3.9	-3.6	-3.3
	Thermal power contribution generation	-2.8	-2.4	-2
	Electricity consumption intensity	-2	-1.6	-1.2

1. Population: According to the press conference on the seventh national population census data in Shanxi Province, the resident population in Shanxi Province has been decreasing in terms of total population. The 2020 census recorded a resident population of 34,905,600 in Shanxi Province, a 2.23% decrease from 2010. The average annual decline is 0.23%, accounting for 2.47% of the country’s total population. The Shanxi Provincial People’s Government announced the 2023 population figures and observed that the province’s population had stabilized. China’s population is expected to expand negatively during the next thirty years, according to the United Nations’ 2019 World Population Prospects report. According to relevant scholars, Shanxi Province’s resident population growth rate is expected to progressively fall in the future following a brief spike [37]. As a result, under the baseline scenario, the rates of change for population growth in the various time periods are set at 0.08 percent for 2021–2025, 0.01 percent for 2026–2035, and -0.04 percent for 2036–2045. The rates of change under the other scenarios are then adjusted in accordance with these values.
2. GDP per capita: In the 14th Five-Year Plan and the Outline of the 2035 Vision, Shanxi Province has proposed that the GDP per capita should reach $20,000 by 2035, and the total economic output should be ranked in the middle of the country. The rate of change of per capita GDP in different periods has been set to achieve this goal. Under the baseline scenario, the rate of change is 6% from 2021 to 2025, 5.5% from 2026 to 2035, and 4% from 2036 to 2045. The rate of change in other scenarios is adjusted accordingly.
3. Carbon Emission Intensity: The completion of the objectives and targets set by the State is the criterion for reducing carbon dioxide emissions per unit of GDP, according to Shanxi Province’s 14th Five-Year Plan. A crucial goal has been established to cut carbon emissions per unit of GDP by 18% by 2025 compared to 2020 in China’s 14th Five-Year Plan and the Outline of the 2035 Vision [38]. The pace at which carbon emission intensity changes in different periods has been set to achieve this. Under the baseline scenario, the rate of change is -4.9% from 2021 to 2025, -4.5% from 2026 to 2035, and -4.1% from 2036 to 2045. The rate of change in other scenarios is adjusted accordingly.
4. Energy Consumption Intensity: The "14th Five-Year" Energy Consumption and Emission Reduction Implementation Program for Shanxi Province aims to decrease the energy consumption per unit of GDP by 14.5% by 2025 compared to 2020. This target aligns with the national standards for reducing energy consumption per unit of GDP set by the "14th Five-Year Plan". The "State Council on issuing the "14th Five-Year" energy saving and emission reduction comprehensive work program notice" further states that China aims to decrease its energy consumption per unit of GDP by approximately 30% by 2030 compared to 2020. To achieve these goals, the rate of change is set at -3% for 2021–2025, -3.5% for 2026–2035, and -4% for 2036–2045 under the baseline scenario.
5. Industrial structure: Because Shanxi Province has abundant coal and mineral resources, a large percentage of its industry is secondary, favoring heavy industry in the industrial structure. According to experts, the characteristics and trend forecast of China’s industrial structure will alter throughout the "14th Five-Year Plan" period, which will end with the secondary industry’s proportion falling to roughly 35.5% [39]. According to the 2050 China Energy and Carbon Emission Report, China will catch up to wealthy nations by that year. The World Bank reports that industrialized countries have more than 70% of their total industrial output in tertiary education, compared to 61% in upper-middle class countries [40].Therefore, the rate of change for the industrial structure is set at -4.1% for 2021–2025, -3.8% for 2026–2035, and -3.5% for 2036–2045 under the baseline scenario.
6. Thermal power generation contribution rate: The "14th Five-Year Plan for Renewable Energy Development in Shanxi Province" outlines the target of achieving 50% of installed capacity and 30% of power generation from new and clean energy sources by the end of the plan. To achieve this, the rate of change for thermal power generation contribution will be set at -3.3% from 2021–2025, -2.9% from 2026–2035, and -2.5% from 2036–2045. Other scenarios will have adjusted rates of change accordingly.
7. Power consumption intensity: This gauges the efficiency of power use by calculating the amount of power used per unit of GDP. During the "14th Five-Year Plan" period, the goal is to keep electricity demand within a reasonable range and gradually shift from "high-speed" to "high-efficiency" electricity consumption. This will optimize the power supply structure and create conditions for the power industry to reach its peak earlier. The rates of change for power consumption intensity under the baseline scenario are set as follows: -2.3% from 2021–2025, -1.9% from 2026–2035, and -1.5% from 2036–2045. The rates of change under other scenarios will be adjusted accordingly.

#### 3.3.3. Prediction results

**Fig 9:**
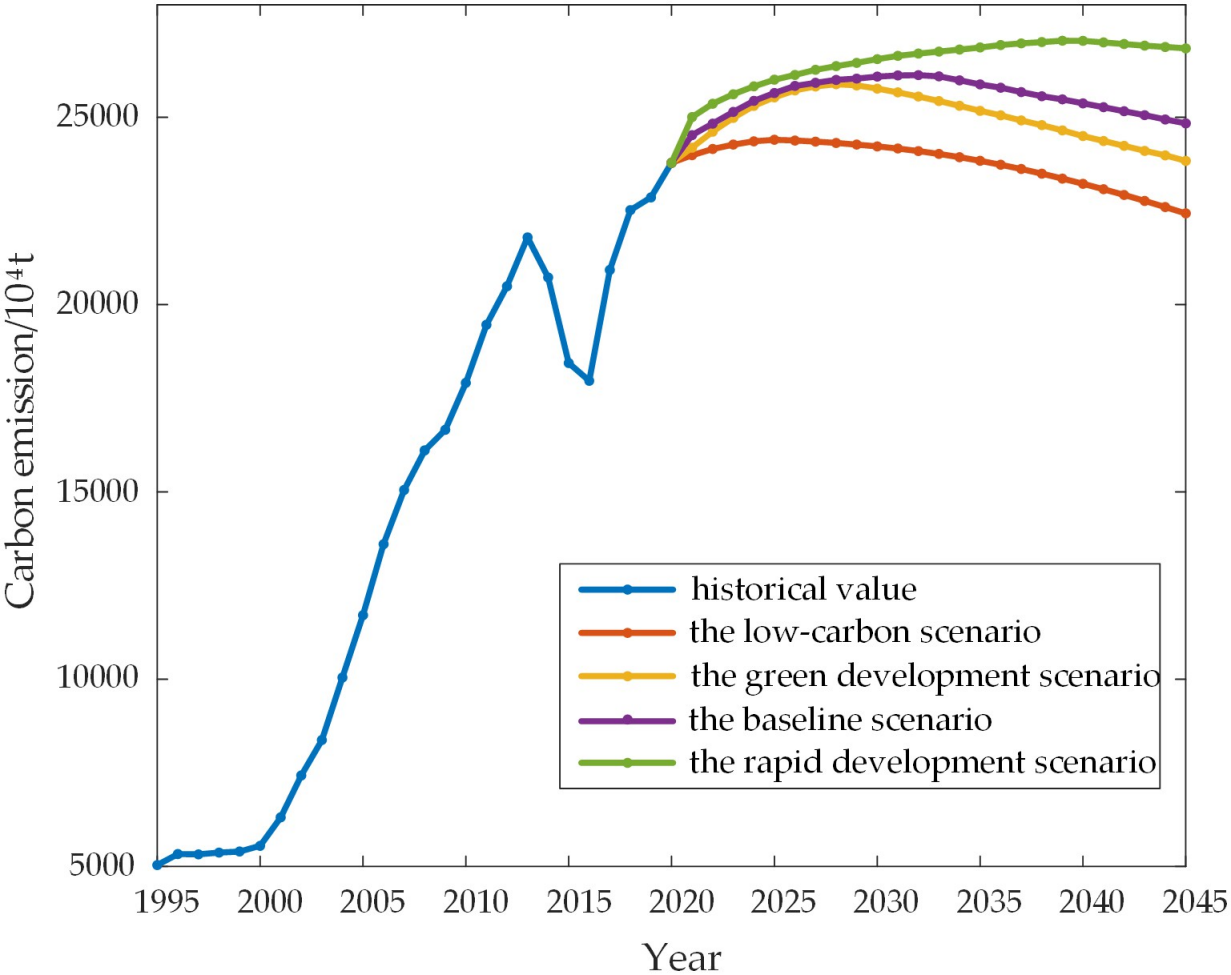
Carbon emission scenarios prediction for power sector in Shanxi Province.

Based on the four different scenario models set and based on the SSA-LSTM model, the carbon emissions from the power sector in Shanxi Province from 2021 to 2045 are predicted. The results under each scenario are shown in Fig 9.

Fig 9 shows that the Shanxi Province’s power sector will reach their peak first in the low-carbon scenario, with the lowest peak amount of carbon. The peak is expected to occur in 2025, with a peak of 243,991,040 tonnes. The peak in the green development scenario will reach 258,828,790 tonnes in 2028. In the baseline scenario, the peak will occur in 2032, with a peak value of 261,246,170 tonnes. In the rapid development scenario, the peak carbon emissions will reach 270,432,400 tonnes in 2039.

Table 6’s parameter settings for the various scenarios show that, in the low-carbon scenario, not only do the population and per capita GDP continue to grow at a medium pace, but also the industrial structure, carbon emission intensity, energy consumption intensity, contribution rate of thermal power generation, and electricity consumption intensity all decrease more quickly. Since Fig 9’s scenario has the earliest peaking time, the power industry in Shanxi Province should concentrate on promoting low-carbon technologies, enhancing energy efficiency, and adjusting the industrial structure going forward in order to encourage the reduction of carbon emissions and see a faster decline in these five indicators. In contrast, the carbon emission intensity, energy intensity, and other variables that are closely linked to the power industry are declining at a medium pace in the Green Development Scenario and the Baseline Scenario, and their peaking speeds are at a medium level. On the other hand, the scenario of rapid development peaks last. Table 6 shows that all the scenario’s components are declining slowly, and since the intensity of energy consumption, the intensity of carbon emissions, and the percentage of the industrial structure have not been adequately managed, the scenario anticipates the latest peak time. This suggests that all energy consumption levels should be moderately controlled during the power industry’s transformation and development process. Excessive carbon emission intensity and an unworkable industrial structure also work against the power industry’s ability to develop sustainably.

## 4. Discussion

The results of this paper show that among the eight influencing factors, per capita secondary industry contribution, industrial structure, and energy structure intensity are the key factors contributing to the carbon emission of electric power in Shanxi, with contributions of 158.79%, 22.70%, and 11.53%, respectively. Electricity consumption intensity, fuel conversion rate, and power structure can significantly reduce carbon emissions, with contributions of -65.19%, -29.31%, and -13.88%, respectively. The biggest positive driver in Li et al.’s analysis of the breakdown of carbon emission drivers in the electricity industry in the Beijing-Tianjin-Hebei region is GDP per capita [41]. According to Jia et al., the main causes of carbon emissions across all industries are economic considerations [42]. Shanxi, as a coal resource-based province, has an industrial structure that favours traditional heavy industry, and there is still a gap between it and developed regions such as Beijing, Tianjin and Hebei, and the secondary industry is still a key factor in the carbon reduction process in Shanxi. Chen et al. found that the power supply structure plays an important role in carbon emission reduction in their study of carbon emissions from the power sector in the Yangtze River Delta [43]. This conclusion is in line with the study’s conclusion in this paper, which states that Shanxi province’s power sector should prioritize encouraging the high-quality grid connection of renewable energy sources like solar and wind power, optimizing the energy structure, enhancing energy use efficiency, and successfully advancing the process of reducing carbon emissions.

According to this paper’s low carbon and green development forecasts, Shanxi Province’s electricity sector would peak in 2025 and 2028, respectively. Because Zhang et al. focused on the power and heat industry, they predicted that Shanxi province will peak in 2029 under the medium development scenario [44]. The present study offers a foundation for investigating carbon reduction strategies in Shanxi Province’s power sector; however, certain limitations remain. Specifically, the study does not currently account for indirect energy consumption in the power sector, which influences peaking rates to some degree. Future research will take into account the effects of indirect emissions on the power industry and carry out more thorough evaluations, which is crucial for the creation of carbon reduction strategies in this industry.

## 5. Conclusions

Determining the factors that influence carbon emissions in the power sector—both positive and negative—and projecting when it will peak are critical for developing effective strategies to reduce carbon emissions. The uniqueness of their energy structure sets resource-intensive cities apart from other cities in terms of their emission reduction strategies. Thus, the carbon emissions of the electric power industry in Shanxi Province are thoroughly examined in this paper, which also chooses pertinent data for carbon measurement from 1995 to 2020. The contribution of each factor in each year is calculated using LMDI, and the peak time of carbon in Shanxi Province is forecast using SSA optimization LSTM and scenarios. The results show that carbon emissions maintain an overall growth trend and slow down in 2016–2020, indicating that the comprehensive reform of the energy revolution in Shanxi Province during the 13th Five-Year Plan period has achieved a stage-by-stage success. With a contribution of 58.79 percent and -65.19 percent, respectively, the secondary industry’s per capita and electricity intensity are the main drivers causing and preventing carbon emissions, and the economy continues to be the main driver. The SSA-LSTM prediction accuracy is much higher than that of the LSTM and BP neural networks, and a reduced error is obtained, according to the comparison of the prediction models. By fine-tuning the LSTM network parameters, SSA dramatically increases the model’s accuracy and offers a solid foundation for future decision-making. With peaks of 243,991,1 and 258,828,8 million tonnes, respectively, the Shanxi power sector can reach carbon peaking under the Low Carbon Scenario and the Green Development Scenario in 2025 and 2028. This means that in the future, the Shanxi power sector will need to concentrate on modifying the industrial structure, enhancing the effectiveness of energy utilization, and facilitating the development of high rates of each of the factors, in order to ensure that the task of reaching carbon peaking will be completed on schedule.

This study closes research gaps on carbon emission in the power sector in energy-intensive regions by building a theoretical framework of the factors influencing carbon emissions in Shanxi’s power sector, identifying the primary influencing factors and the peak time under various scenarios, and serving as a crucial foundation for relevant policy formulation and real-world investigation. The findings of this study suggest that when developing strategies to reduce carbon emissions, Shanxi Province’s power sector should focus more on the impact of the secondary industry, energy structure, power consumption, and energy utilisation. Additionally, it should leverage the correlation between each of these factors and carbon emissions to effectively encourage reduction of carbon emissions. Aside from encouraging public participation in energy emission reduction initiatives, pertinent departments should also aggressively educate the public about low-carbon living and climate change, encourage green energy consumption, and help people adopt green lifestyles. This study focuses on the carbon emissions of Shanxi Province’s electric power industry in order to shed light on the primary driving variables and emission reduction goals. Future research will centre on the examination of carbon emissions in resource-intensive provinces after a more thorough analysis is completed. The elements that reduce carbon emissions are compiled based on the features of regional growth, and the peak period is determined using accurate forecasting techniques. For the purpose of encouraging the industrial adjustment and economic transformation of the energy provinces, investigating the emission reduction path on this premise is extremely important.

## 6. Policy recommendations

Analysing the results of the study, it can be seen that the proportion of the secondary industry and the intensity of electricity consumption have a more significant impact on the carbon emissions of Shanxi’s power industry. The government’s Shanxi Carbon Peak Implementation Plan states that future development should support coal’s transition from a fuel to a finished good in order to optimize the industrial structure and gradually develop into a product with a high rate of carbon sequestration. Shanxi Province’s share in secondary industry has decreased recently as a result of the policy’s direction. The industry’s digital and intelligent transformation should be encouraged in order to empower information, foster the synergistic development of the industrial chain’s upstream and downstream, and accelerate the process of reducing carbon emissions. To successfully encourage the power industry’s carbon peak, appropriate action must be taken to increase energy efficiency and regulate the intensity of power consumption. According to Shanxi Province’s 14th Five-Year Plan, by 2025, electricity will make up 40% of end-use energy consumption. For Shanxi Province to achieve negative growth in coal consumption and develop a low-carbon, green power system, it needs to optimize its structure for energy consumption, enhance the replacement of clean energy, and fully capitalize on the benefits of natural gas resources and industry. Based on this, the relevant departments ought to increase their research and development expenditure in science and technology innovation, concentrating on important technologies in important fields like nuclear power, energy storage, hydrogen energy, clean and efficient coal use, and carbon capture and storage. They should also increase their support for Carbon Peak through science and technology innovation.

The study presented in this paper enhances the carbon peak path reference for energy-intensive locations. Based on their energy kinds and resource advantages, energy-intensive regions should encourage the transformation and reform of resource-based economies. Shanxi and Northeast industrial zones are examples of coal-intensive regions that should concentrate on supply-side changes to increase the output energy’s quality and efficiency. To facilitate the export of electricity, increase the outgoing corridors’ energy utilization efficiency. Establishing a clean power supply base with wind, fire and storage energy complementing each other on the premise of guaranteeing a secure and stable supply of energy. The western region and Xinjiang, which are places with a substantial amount of green energy, ought to concentrate on coordinating supply and consumption sides of the equation and encourage the best possible distribution and use of green energy across the nation. Strengthen inter-regional energy cooperation and steadily advance the energy revolution process with the aid of the regional energy revolution.

## Supporting information

S1 File (DOCX)
