# Supplementary material for: Study on carbon emission driving factors and carbon peak forecasting in power sector of Shanxi province
Source: PLoS One. 2024 Jul 12;19(7):e0305665. doi: 10.1371/journal.pone.0305665 (PMC11244784; doi:10.1371/journal.pone.0305665)
Supplement: S1 File — (DOCX) [file pone.0305665.s001.docx]

Appendix

function [Best_pos,Best_score,curve]=SSA(pop,Max_iter,lb,ub,dim,fobj)

ST = 0.6;%

PD = 0.6;%

PDNumber = round(pop*PD);

SDNumber =round( pop - pop*PD);

X0=initialization(pop,dim,ub,lb);

X = X0;

fitness = zeros(1,pop);

for i = 1:pop

fitness(i) = fobj(X(i,:));

end

[fitness, index]= sort(fitness);

BestF = fitness(1);

WorstF = fitness(end);

GBestF = fitness(1);

for i = 1:pop

X(i,:) = X0(index(i),:);

end

curve=zeros(1,Max_iter);

GBestX = X(1,:);

X_new = X;

for i = 1: Max_iter

BestF = fitness(1);

WorstF = fitness(end);

R2 = rand(1);

for j = 1:PDNumber

if(R2<ST)

X_new(j,:) = X(j,:).*exp(-j/(rand(1)*Max_iter));

else

X_new(j,:) = X(j,:) + randn()*ones(1,dim);

end

end

for j = PDNumber+1:pop

if(j>(pop - PDNumber)/2 + PDNumber)

X_new(j,:)= randn().*exp((X(end,:) - X(j,:))/j^2);

else

A = ones(1,dim);

for a = 1:dim

if(rand()>0.5)

A(a) = -1;

end

end

AA = A'*inv(A*A');

X_new(j,:)= X(1,:) + abs(X(j,:) - X(1,:)).*AA';

end

end

Temp = randperm(pop);

SDchooseIndex = Temp(1:SDNumber);

for j = 1:SDNumber

if(fitness(SDchooseIndex(j))>BestF)

X_new(SDchooseIndex(j),:) = X(1,:) + randn().*abs(X(SDchooseIndex(j),:) - X(1,:));

elseif(fitness(SDchooseIndex(j))== BestF)

K = 2*rand() -1;

X_new(SDchooseIndex(j),:) = X(SDchooseIndex(j),:) + K.*(abs( X(SDchooseIndex(j),:) - X(end,:))./(fitness(SDchooseIndex(j)) - fitness(end) + 10^-8));

end

end

for j = 1:pop

for a = 1: dim

if(X_new(j,a)>lb(a))

X_new(j,a) =lb(a);

end

if(X_new(j,a)<ub(a))

X_new(j,a) =ub(a);

end

end

end

for j=1:pop

fitness_new(j) = fobj(X_new(j,:));

end

for j = 1:pop

if(fitness_new(j) < GBestF)

GBestF = fitness_new(j);

GBestX = X_new(j,:);

end

end

X = X_new;

fitness = fitness_new;

[fitness, index]= sort(fitness);

BestF = fitness(1);

WorstF = fitness(end);

for j = 1:pop

X(j,:) = X(index(j),:);

end

curve(i) = GBestF;

end

Best_pos =GBestX;

Best_score = curve(end);

end

Supporting information

**S1 Fig.** **Structure of LSTM model.**

**S2 Fig.** **The steps of SSA-LSTM modelling.**

**S3 Fig.** **Carbon Emissions from Power Sector in Shanxi Province, 1995-2020.**

**S4** **Fig. Cumulative contribution of carbon emissions from each factor.**

**S5 Fig.** **Decomposition of the value of contributions.**

**S6 Fig. Comparison of SSA-LSTM predicted and true values.**

**S7 Fig. Comparison of LSTM predicted and true values.**

**S8 Fig. Comparison of BP predicted and true values.**

**S9 Fig. Carbon Emission Scenarios Prediction for Power Sector in Shanxi Province.**

**S1 Table. Minimum data set of factors influencing carbon emissions.**

**S2 Table**. **Table of descriptive statistics for the smallest data set.**

**S3 Table**. **Description of model variables.**

**S4 Table. A breakdown of the contribution of each factor.**

**S5 Table. Comparison of the prediction accuracy of the three models SSA-LSTM, LSTM, and BP.**

**S5 Table. Parameter settings for different scenarios.**

**The minimal data set:**

| **Year** | **Carbon emissions**  **/tons** | **Population**  **/million** | **Gross regional product**  **/million dollars** | **Energy consumption**  **/tons** | **Total output value of the secondary industry/ million dollars** | **Thermal power generation**  **/billion kWh** | **Total power generation**  **/billion kWh** | **Electricity consumption**  **/billion kWh** |
| --- | --- | --- | --- | --- | --- | --- | --- | --- |
| 1995 | 5037.76 | 3077.28 | 10760300 | 28603900 | 4944500 | 498.85 | 505.97 | 399.16 |
| 1996 | 5331.21 | 3109.26 | 12921100 | 30365000 | 6002100 | 519.52 | 526.89 | 431.22 |
| 1997 | 5323.62 | 3140.89 | 14760000 | 30025200 | 7075800 | 540.26 | 546.02 | 446.01 |
| 1998 | 5369.93 | 3172.20 | 16110800 | 30191800 | 7612500 | 548.02 | 554.03 | 440.31 |
| 1999 | 5398.39 | 3202.63 | 16671000 | 30228400 | 7854700 | 558.45 | 569.83 | 459.34 |
| 2000 | 5550.08 | 3247.8 | 18457200 | 31273100 | 8583700 | 607.27 | 620.31 | 506.09 |
| 2001 | 6310.78 | 3271.63 | 20295300 | 35887000 | 9560100 | 691.14 | 710.33 | 557.08 |
| 2002 | 7429.73 | 3293.71 | 23248000 | 41729500 | 11343100 | 823.18 | 842.01 | 628.83 |
| 2003 | 8374.33 | 3314.29 | 28542500 | 47376200 | 15207300 | 945.71 | 965.01 | 731.77 |
| 2004 | 10042.85 | 3335.07 | 34959900 | 61361400 | 19306300 | 1058.00 | 1078.99 | 841.55 |
| 2005 | 11707.92 | 3355.21 | 40793800 | 82880528 | 23895100 | 1291.65 | 1311.97 | 892.46 |
| 2006 | 13603.57 | 3374.55 | 47136000 | 91323672 | 27992800 | 1502.50 | 1526.37 | 1036.06 |
| 2007 | 15050.57 | 3392.58 | 59355800 | 95792354 | 36023100 | 1713.18 | 1760.50 | 1269.69 |
| 2008 | 16106.66 | 3410.64 | 72229800 | 105723382 | 43889500 | 1764.93 | 1793.78 | 1238.25 |
| 2009 | 16657.72 | 3427.36 | 71476100 | 106268532 | 40912000 | 1848.76 | 1873.80 | 1196.24 |
| 2010 | 17909.42 | 3574.11 | 89039000 | 108815323 | 53496400 | 2104.00 | 2151.00 | 1381.25 |
| 2011 | 19460.40 | 3562.37 | 108944100 | 118473448 | 67501500 | 2301.73 | 2343.97 | 1576.23 |
| 2012 | 20488.06 | 3548.21 | 116831100 | 124623103 | 68527000 | 2456.14 | 2545.91 | 1672.39 |
| 2013 | 21793.38 | 3534.98 | 119872300 | 125720787 | 66843200 | 2551.32 | 2641.11 | 1731.48 |
| 2014 | 20724.59 | 3528.49 | 120947100 | 126221203 | 63779600 | 2546.03 | 2647.02 | 1726.14 |
| 2015 | 18438.26 | 3518.62 | 118363900 | 124151935 | 52196500 | 2330.26 | 2449.00 | 1644.09 |
| 2016 | 17964.01 | 3514.48 | 119464000 | 127100909 | 51136200 | 2362.85 | 2535.00 | 1706.94 |
| 2017 | 20924.11 | 3510.46 | 144842700 | 128289160 | 66353300 | 2607.22 | 2861.00 | 1893.59 |
| 2018 | 22521.87 | 3502.47 | 159581300 | 126479434 | 70744600 | 2853.44 | 3203.00 | 2175.43 |
| 2019 | 22862.42 | 3496.88 | 169616100 | 132648156 | 74663000 | 2960.80 | 3362.00 | 2233.63 |
| 2020 | 23783.14 | 3490.50 | 176519300 | 131285167 | 76754400 | 3032.50 | 3504.00 | 2343.49 |
